# Supplementary material for: Probing the conserved roles of cut in the development and function of optically different insect compound eyes
Source: Front Cell Dev Biol. 2023 Mar 31;11:1104620. doi: 10.3389/fcell.2023.1104620 (PMC10102356; doi:10.3389/fcell.2023.1104620)
Supplement: Supplementary file 1 [file Table1.DOCX]

Supp Table 1. *cut* gene sequence identified from *T. marmoratus* transcriptome and the forward and reverse primer sequences used for amplifying the *cut* dsRNA amplicon.

| *cut* gene sequence 5’-3’ | |
| --- | --- |
| GATGCTGTCCAAAAGATACCAACGCCAATACAAGCTGATTCTACCGAAAAATCTACTGCTTCTACCCCCGTACCTGAACAAACTGGAAGTCAGCCATCACCTCAACCTTCCCCGTTGATCAACGGGAATCCGAAGAGTCCAGAGGATAACAACAACGTACACGTGTCCAACAACAATATGAACCCACTCAACGCCGCAGCGATGTTATCCGTGAACAACTTCCTGAGGAACGACGATTCGCTGAAGAGCCCTTACCGATTCGACGACCATAGATCACCGTTCAGATTCGCCGACGAACTCGGTATGCCTGGCGCAATGGTAGGGAGACTAGGAGAAAGCTTAATCCCCAAAGGTGACCCAATGGAAGCAAGGCTGCAAGAGATGCTCCGCTATAACATGGATAAATACGCAAACCAAAACCTGGACACGCTGACAATCTCCAGAAGGGTTCGCGAACTCCTGTCCATACATAACATCGGACAAAGACTATTCGCTAAGTACATCCTGGGACTATCCCAGGGGACTGTTTCTGAGCTGCTGTCGAAGCCCAAGCCCTGGGATAAGTTGACTGAGAAGGGACGGGACAGCTACAGGAAGATGCACGCATGGGCATGTGACGAGAATTCTGTCATGGTTCTCAAATCGCTTATACCAAAAAAAGGTAAAGACACAACAATCCCCCCCTTCGGTCGACCCGAACCCGACATCACCGAGGAGAGGATCGCCCACATCCTCAACGAAGCCACCAGTCACATGATGAAGTCGAACACTGAAGATTCGAGGAGCAACGATGACAGCAAAAGCCCGTCTCAAGGACAGTGCCCAAGTCCCTTCTCCAAGGATTCTTCACAAAACAGAAGATTGAAGAAATACGAAAACGATGATATACCTCAAGAAAAAGTAGTGAGAATATATCAAGAAGAATTAGCCAAGCTAATGGGAAGAAGAGTTGAAGATATGAGGAATCCAAGAGATCACTTTTCAAGTATCTTCTTCCCCCAATTCTTGAGCGGCGGTCTTCCAATGGACAGAACCCAAGAAGAAATCCGGATGGCGTTAGACGCTTACCACAGAGAGCTGAGTAAGCTGAACCAGTGTCAGAATCCAGCTCAGCTTCCAAACCTCCCAGGTCTTCCCGGTCTCCTGGCCCTCCAGCAGCAAGCTATGGTCCAGAACCCCCAACCTCAGAACGGCGGCGTCCAAGACTTATCCCTACCAAAAGACAACAAACCAAAGAGCATCAACGGCATGGAAGACGCGAAAGAAAAAGAAGCCATGGATGAAGCCATGAAACACGCCGGCAGCGCGTTCAGCTTGGTCCGACCAAAACTAGAACCAGGAACCCAACAAGCAAGTTCCACAGCTTCAAGTCCGCTAGGCAACTCCATTCTTCCACCTGCGATGACCCCAACAGATGACTTCACCTCATCAGCAGCCGCAAGCCCCTTGCAGAGAATGGCTTCCATTACAAACTCACTGATCTCTCAACCACCGACCCAATCCCACCACAGTCCGGCACAGCGACCTCTCAAAGCAGTTCTTCCTCCGATCACCCAACAACAGTTTGATCTCTACAACAACCTCAACACAGAAGACATCGTCAAGAAAGTAAAAGAACAACTCAGTCAGTATTCCATCAGCCAGCGGTTATTCGGAGAAAGTGTCCTGGGTCTTTCGCAAGGATCAGTCAGTGACCTCCTTGCGAGACCCAAGCCCTGGCACATGCTCACCCAGAAAGGACGCGAACCTTTCATCAGGATGAAGATGTTCTTAGAAGATGAAAACGCAGTCCACAAACTGGTAGCCAGTCAGTACAAAATCGCACCCGAAAAACTCATGAGAACCGGCGGATACGGCACTTCCACCTCAGCTCTTGCCAAACCAATGCCACCCACGCCAAAAATGATCAGCGAAGCCGCCGGATTACTGAACAAAATGCAACAAGAAGGCCAAAATGCTCTTCTCCCTCCGAGCCTGAACCTTGGACCCCCTGGACAACAAAATCTTCCACAACCACCTCCCCCACCCATGTTGTTAACTCCTCCAGGAATCTCACCACATCATTCCATGTCCATGAAATCCGCCCAAGAACAAATGAAACAACAAAATCAAAGTCCTCATCCCGGAGTCCCCGTCTCACCGATGGGACAACAACCATCCTCCGCCATGAGAAGTCTCCACCAACACATATCCCCAAGCGTCTACGAGATGGCAGCGCTAACACAAGACTTGGATACTCAAGTGATCACCACCAAGATCAAAGAAGCTTTACTGGCCAACAATATTGGGCAAAAGATATTCGGGGAGGCCGTGCTGGGTCTTTCGCAGGGATCAGTGAGCGAGTTGCTCTCAAAACCCAAACCTTGGCACATGTTGAGCATAAAGGGTCGCGAACCGTTCATCAGGATGCAACTTTGGTTGAACGATGCTCATAACGTAGACCGCCTACAAGCACTGAAAAACGAAAGACGAGAAGCCAACAAAAGACGAAGATCTTCTGGTCCAGGAGCTCACGATAACAGCTCGGACACGTCATCGAACGACACATCTGAGTTCTACCACTCCAACTCTCCAGGACCCGGGCCTCCATCCGCCAAGAAGCAGCGCGTGCTCTTCTCCGAGGAGCAGAAGGAAGCCCTCAGGTTGGCGTTCGCGTTAGATCCGTACCCCAACGTGGCGACCATCGAGTTCCTGGCTGGCGAACTGGCCCTCAGCAGTAGGACCATCACCAACTGGTTCCACAACCACCGTATGAGACTAAAGCAACAAGTCCCCCACGGAATGCCCTCCGACATCCCGCCGAGAGACCAAAACAGCGGACAGACACCGTTCGATCCAGTACAATTTAGACTGTTATTAAACCAAAGATTATTAGATCTATCTAAAGAAAGGATGGGTCTAGGAGGAGTACCTCTACCCTACCCACCTTACTTCGCCGCCAACTCGAATCTGGCGGCGTTGATCAGCAGAGGCCTGATCCCGTCGGGCGACGTAGATCTCTCCGCTCTCGGTAACGCCGTGAAGGAGCAGATGAGCGGTCTGGATCTCTCCATGGGCTCTTCCTTAAAGCACGAACCTTCCGGCGACTACGAGGACGACGAAGACGACGTAGAAAGTAACATGGGATCAGAAGATTCCGATAACATGTCAGCATCAAGTTTAACCCACGGCGAAGTTAAAGTAGAACAAAAGGTCACCGGCCCCGTACCGAGAAGTTCTCGAAGAAAACCCGCCGCACCGCAGTGGGTCAACCCGGAGTGGCAAGACGAAAACAAAGAAGCCAAAGCTCCGACAGGAGACGAGGTAATAATCAACGGAGTATGCGTGATGCAAACGGAAGACTACGGCAGACGGAATTCGGAAGAGACAGTTCGGGTGGAACCTCAAGCGGTCATGGACCGGTTCGACGACGACGCGAGCGACGCGTCGTCGTCGGTGAGCCATGACGAGAACGCGGACCGTCGAAGCCCTTCTGCTCAAGTCAAACAAGAGAGAGAAGAACCTGAAGAAATAGTTACGAGACAAAGTAGTGATGAACAAATCGAACACACAGACAAACAAATTAAAACAGAGAACGAGGAAGAAAGATGGGAATATTAG | |
| Forward: CACCGTTCGATCCAGTACAA | Reverse: CGTTTGCATCACGCATACTC |
