# Supplementary material for: Probing the conserved roles of cut in the development and function of optically different insect compound eyes
Source: Front Cell Dev Biol. 2023 Mar 31;11:1104620. doi: 10.3389/fcell.2023.1104620 (PMC10102356; doi:10.3389/fcell.2023.1104620)
Supplement: Supplementary file 2 [file DataSheet1.docx]

**Supplementary figures**


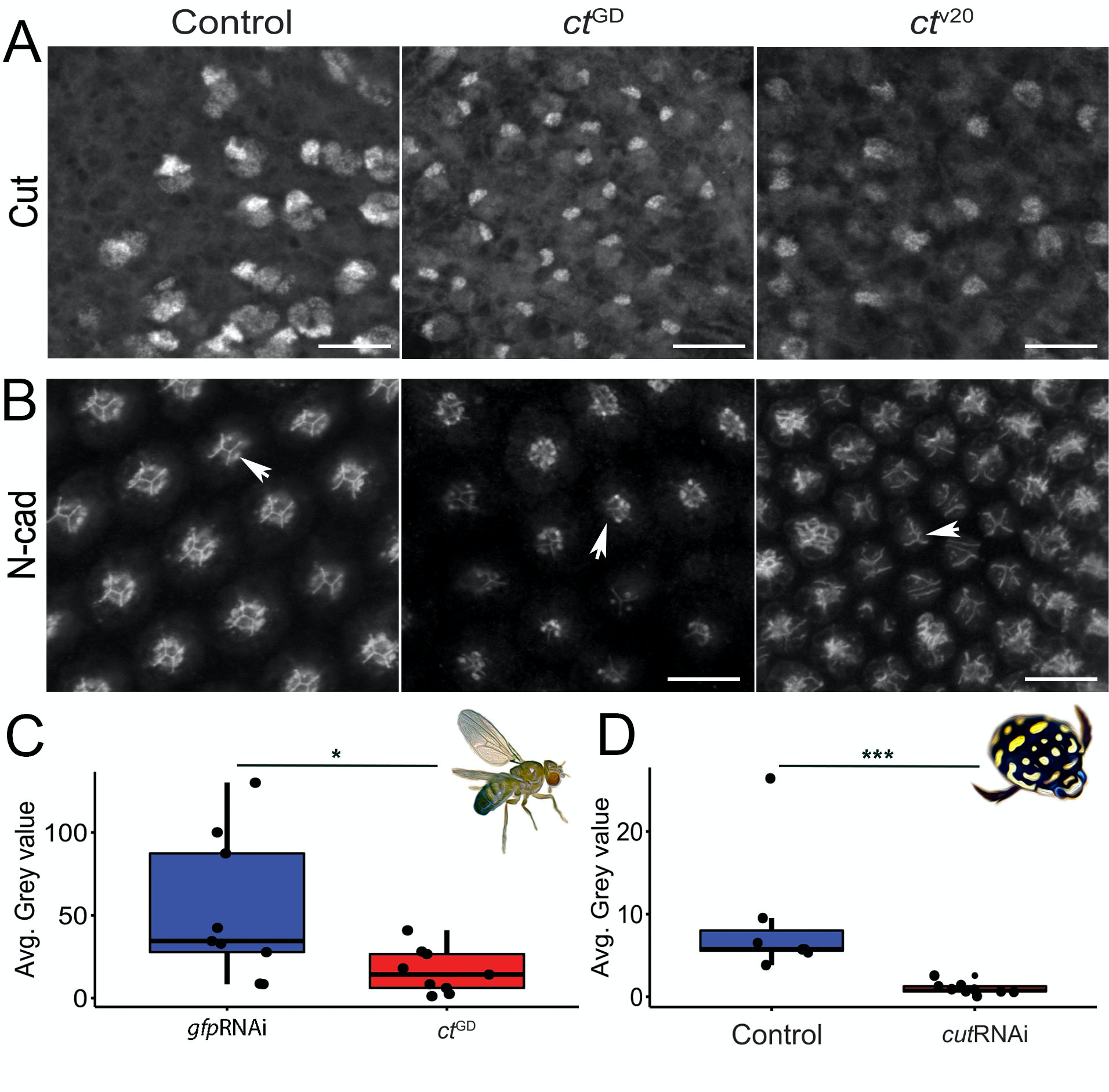


Supp Fig. 1. Confirmation of SC-specific *cut* knockdown in developing *D. melanogaster* eyes with knockdown-specific irregularities in the tetrad organization. A. To verify that *cut* knockdown was SC-specific, we also imaged Cut-positive sensory bristle nuclei and found comparable Cut immunoreactivity in all three fly lines. B. N-cadherin immunoreactivity illustrates that the control retinas show the typical “H” organization of the apical surfaces of the four SCs, whereas this organization is inconsistent or lost in both *ct*^GD^ and *ct*^V20^ retinas (arrows). Scale bars = 10 µm. Successful *cut* knockdown in SCs was qualitatively confirmed using Cut antibody staining and grey value measurements. Average grey values of the SCs from C) 37% developed retinal wholemounts of *gfp*RNAi flies (blue) were significantly higher than *ct*^GD^ flies (red) (n=9 each, p = 0.019* based on a Wilcoxon’s Rank sum test) and; D) early retinal wholemounts of control beetles (n=7,blue) were significantly higher than the *cut*RNAi beetles (n=9,red) (p = 0.00017 *** based on a Wilcoxon’s rank sum test).


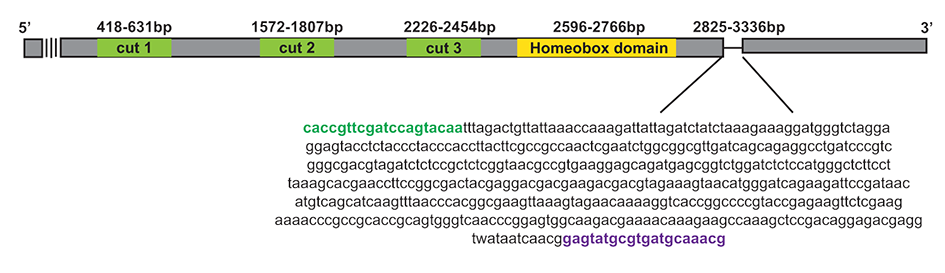


Supp Fig. 2. *Cut* gene in *T. marmoratus* and *cut*RNAi knockdown sequence. *Cut* is characterized by three specific domains (indicated as cut 1, 2, and 3) and a homeobox domain. The RNAi knockdown sequence is located toward the 3’ end of the homeobox domain. The primer binding regions are indicated in green (forward) and purple (reverse).


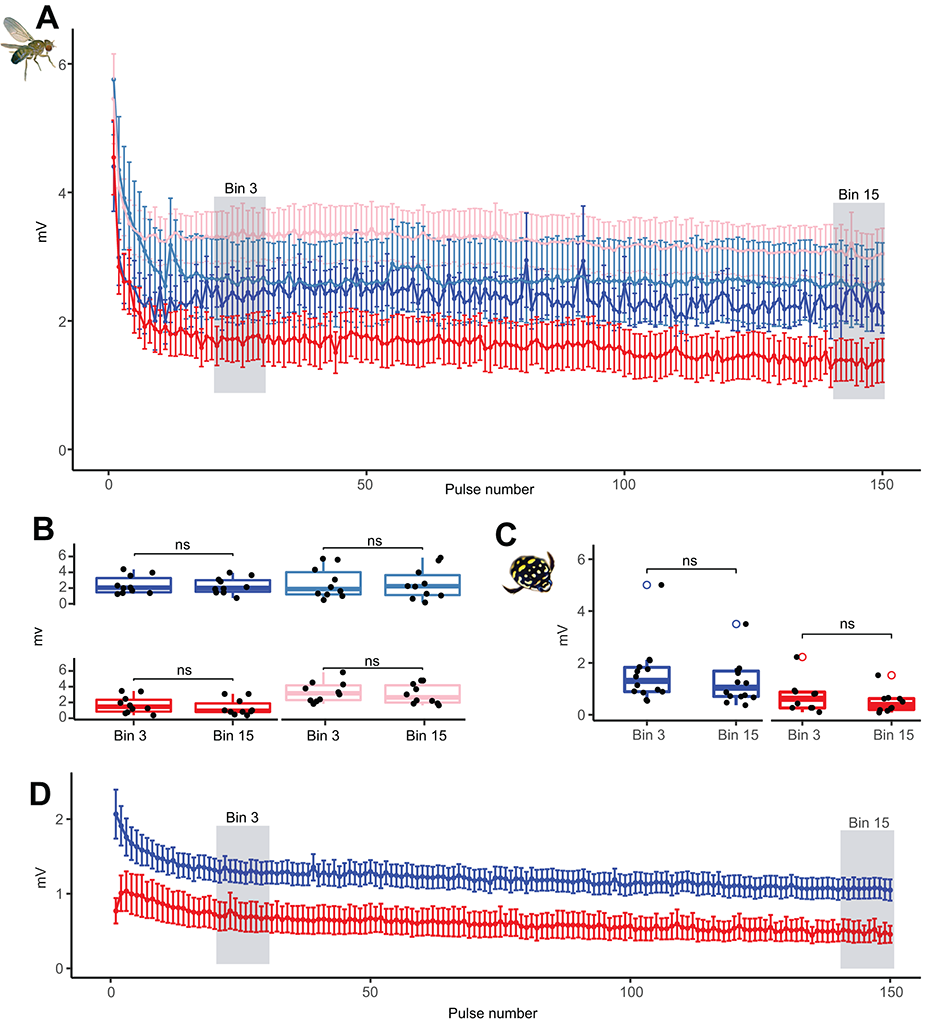


Supp Fig. 3. Electroretinograms for testing the sustainability of the photoreceptor response in both species. A. The average PR response (with standard error) of *D. melanogaster* to 150 pulses exhibits an initial decline (due to adaptation) followed by a somewhat lower signal than was sustained in all four lines throughout the pulse series. n = 10. B. Comparison between the responses of the third and last bins in *D. melanogaster*. C. Comparison between the responses of the third and last bins in *T. marmoratus*. D. PR response (with standard error) of *T. marmoratus* to 150 pulses, illustrating a sustained signal (after initial adaptation) in both control and *cut*RNAi individuals.
